# Outcomes for Degenerative Cervical Myelopathy Following Implementation of the AO Spine International Guidelines: A Single-Centre Service Evaluation

**DOI:** 10.1177/21925682241301049

**Published:** 2024-11-10

**Authors:** Jamie Brannigan, Sundar K. Vellaiyappan, Oliver D. Mowforth, Joseph Magee, Jibin J. Francis, Benjamin M. Davies, Mark R. Kotter

**Affiliations:** 1 Division of Neurosurgery, Department of Clinical Neurosciences, Addenbrooke’s Hospital, 2152 University of Cambridge, Cambridge, UK

**Keywords:** cervical, myelopathy, spondylosis, spondylotic, stenosis, disc herniation, ossification posterior longitudinal ligament, degeneration, disability, recovery, outcomes, spinal cord compression

## Abstract

**Introduction:**

Degenerative cervical myelopathy (DCM) is a syndrome of symptomatic cervical spinal cord compression due to degenerative spinal changes. Until recently there was no formal consensus on exactly which patients are suitable for surgical or conservative management. The AO Spine international guidelines were introduced to address this issue, based on the best available current evidence. However, their implementation into routine clinical practice has not yet been reported. The objective was to evaluate surgical outcomes following the implementation of the AO Spine guidelines at our spinal neurosurgical centre.

**Methods:**

A service evaluation was conducted using data collected from electronic healthcare records for 259 patients, with outcomes of interest including change in mJOA score and complications. Data from 193 patients were included in the final analysis.

**Results:**

There was a mean improvement of the mJOA score by 1.53 points, 1.44 point and 1.92 points at post-operative intervals of 3 months (*P* < .001), 6 months (*P* < .001) and 12 months (*P* < .001). The percentage (number) of patients whose increase in mJOA score was greater than or equal to the minimal clinically importance difference (MCID) was 41% (44/107), 34% (33/96) and 43% (49/114) at these respective time intervals. Intraoperative complications occurred in 28 patients (11.7%). No association was found between BMI and postoperative change in mJOA score.

**Conclusion:**

Our results are comparable to those from best practice data and suggest adherence to international guidelines provides a service that promotes meaningful recovery for patients with DCM. Therefore, our results offer support for implementation of the AO Spine international guidelines in clinical practice.

## Introduction

Degenerative cervical myelopathy (DCM) is a syndrome of symptomatic cervical spinal cord compression due to degenerative spinal changes.^1^ Examples of such changes include ligamentous hypertrophy or ossification, disc herniation and osteophytosis.^2-4^ DCM is diagnosed by the presence of myelopathic clinical signs and symptoms in combination with radiological evidence of degenerative cervical cord compression.^5,6^ Delays in diagnosis are common while progressive spinal degeneration causes increasing, and frequently irreversible, neurological disability.^7,8^ DCM has one of the worst quality of life scores of any chronic disease.^9-11^

DCM is the most common cause of non-traumatic spinal cord injury in adults worldwide^2^ and is highly underrecognized.^12,13^ In published clinical series, the incidence and prevalence in North America are suggested to be 41 and 605 per 1,000,000, respectively.^14^ However, these are likely to be underestimates limited by poor and incomplete data. For example, one study reported DCM in 18% of hip fracture patients^12^ and an imaging study reported 59% of randomly recruited individuals had radiological evidence of cord compression and 1% had DCM.^15,16^

Presenting clinical features are highly variable and often subtle or non-specific^17,18^ but commonly include pain, numbness and paraesthesia, gait instability and falls, loss of manual dexterity, autonomic features such as urinary retention and incontinence, pyramidal weakness and upper motor neuron signs.^1,19,20^ The diagnosis should be considered in any patient over the age of 40 presenting with progressive neurological symptoms and is a mandatory differential to consider in patients of this age group with clinical features suggesting a bilateral carpal tunnel syndrome or cervical spondylotic radiculopathy.^16^

Management options for DCM are non-operative or surgical.^21^ Currently, surgical decompression is the only treatment backed by high-quality evidence^22,23^; it can halt but often not reverse existing neurological deficits.^24,25^ This evidence-base was recently consolidated by AO Spine to inform international guidelines; surgical management is recommended for patients with moderate (mJOA score 12-14) or severe (mJOA score < 12) DCM and either surgery or a supervised trial of structured rehabilitation was recommended for patients with mild (mJOA score > 14) DCM. Patients initially managed non-operatively should be offered surgery if their condition deteriorates or fails to improve.^26^ Whilst the guidelines make recommendations about when to offer surgery, they do not address the type of surgery that should be offered; this is left to the discretion of the treating surgeon. Furthermore, to our knowledge, the implementation of these guidelines in routine practice has not yet been reported.

The aim was to evaluate surgical outcomes following introduction of the AO Spine guidelines into clinical practice at a single neurosurgical spinal centre. The objective was to consider the demographics and surgical details, outcomes and complications, with results compared to those from published international cohorts.

## Methods

### Design

A single centre service evaluation was conducted and is reported following the Equator Network Strengthening the Reporting of Observational Studies in Epidemiology (STROBE) guidelines.^27^

### Approval

The work was approved by Cambridge University Hospitals Audit Team (ID5785/PRN11785).

### Setting

Patients attending a specialist spinal clinic at Cambridge University Hospitals NHS Foundation Trust between 1st June 2016 and 22nd November 2022 were included in the study. Although the AO Spine guidelines were published in 2017, MRK was a member of the guidelines development committee and had thus began to implement the guidelines in his clinical practice prior to 1st June 2016. Data for all patients included in the evaluation were extracted from electronic healthcare records that were created as part of routine clinical care. No additional data were collected or stored specifically for this work.

The range of durations between operation and the first 3-months post-operative clinic appointment was 1-4 months with all but one patient were assessed between 2 and 4 months. The range of durations between the operation and the 6-months post-operative clinic appointment was 5-11 months, whilst the range of durations between the operation and the 12-months post-operative appointment was 10-23 months. For all patients, the 12-months appointment was chronologically later than the 6-months appointment, despite the overlap in ranges for the cohort as a whole.

### Participants

Patients with DCM attending clinic were eligible for inclusion in the study. Most patients initially presented following referral from a general practitioner to a musculoskeletal triage service or to neurology. All patients included were under the care of a single consultant spinal neurosurgeon (MRK). The first preoperative clinic appointment was defined as the first documented consultation in a spinal surgery clinic for DCM. Having undergone multiple operations for DCM did not exclude patients from the study and the first operation for which baseline and postoperative modified Japanese Orthopaedic Association (mJOA) scores were available was used as the surgery of reference.

### Variables

Data was collected from preoperative clinic appointments, perioperative hospital admission and postoperative clinic appointments. Variables of interest included demographic data such as age, gender and body mass index. Disease-related variables included symptoms, physical examination findings, most recent preoperative mJOA score, comorbidities, source of referral to clinic, radiological findings, number of levels of symptomatic spinal cord compression, history of DCM, history of surgery for DCM, history of physiotherapy for DCM and current management plan. Perioperative variables of interest included: American Society of Anaesthesiologists (ASA) score,^28,29^ operation type, intraoperative complications, receipt of postoperative physiotherapy, mJOA score on discharge and requirement for readmission to hospital.

The primary outcome measure of interest was change in mJOA score following surgery.^30^ Preoperative and postoperative mJOA scores were compared. Where any comparison is made between preoperative and postoperative mJOA scores, the former refers to the most recent preoperative mJOA score.

The overall minimum clinically important difference (MCID) for the mJOA score depends on DCM severity. For baseline severities of mild, moderate and severe, the MCID is considered to be one, two and three points, respectively.^31,32^ Our calculated MCIDs incorporated this stratification, with respect to the most recent preoperative mJOA score.

### Data Sources

All data were collected from electronic healthcare records.

### Study Size

A sample of 259 DCM clinic patients between 1st June 2016 and 22nd November 2022 were included in the study.

### Quantitative Variables

The mJOA is an 18-point score of DCM severity. Scores for mild (15-17), moderate (12-14) and severe (≤11) are well-established in the literature.^33^ These groupings were utilised in this study. Pre-operative mJOA was defined as the most recent mJOA before surgery. Rounded numbers are given to the nearest integer in all tables, unless otherwise specified.

### Statistical Methods

Three separate analyses were conducted based on 107 (3 months), 96 (6 months) and 114 (12 months) pairs of preoperative and postoperative mJOA scores; an additional three analyses were conducted for mJOA subcomponent scores. Shapiro-Wilk and Kolmogorov-Smirnov tests along with visual inspection of histograms and QQ plots were used to test for normality of distribution and the conclusion was made that data were not normally distributed. Transformation of the data proved unhelpful. For these reasons, the sign test was used to assess significance. The sign test with a continuity correction was used for the 3-month total mJOA score analysis and all other analyses used the exact sign test. Prism Version 9 (GraphPad, Boston, United States) was used for analysis, with significance set at *P* < .05. Unless otherwise stated, we report mean values (standard deviation).

## Results

### Participants

**Figure 1.**
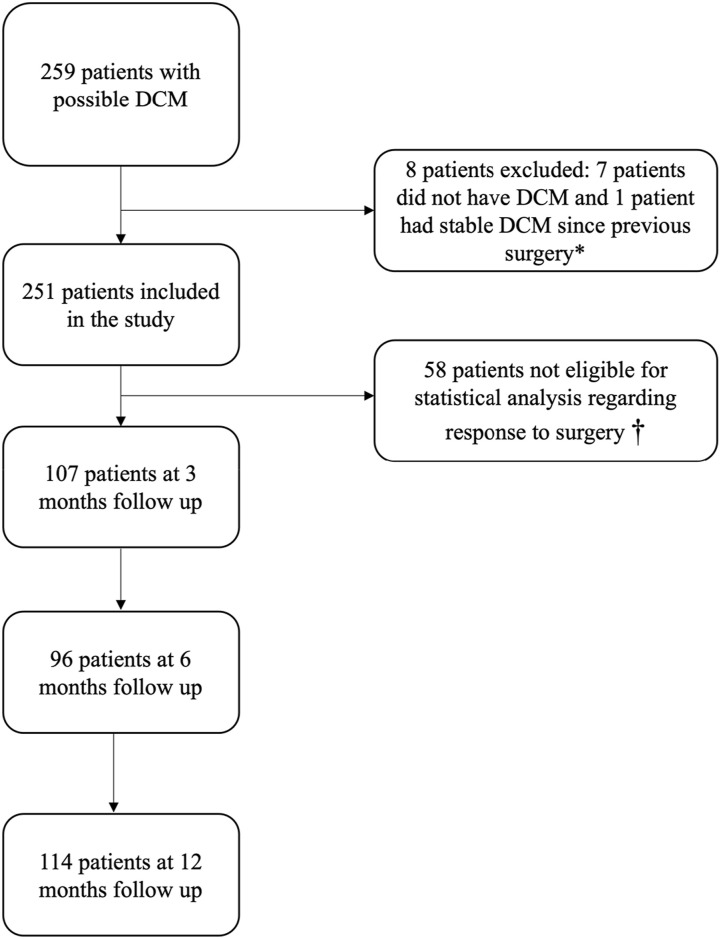
Flowchart of patient selection. † Of these 58 patients, 12 did not have surgery, 20 did not have pre-operative mJOA scores recorded, 16 did not have post-operative mJOA scores recorded and 11 were participating in the RECEDE-Myelopathy trial or IMAGE DCM trials.

Of the total 259 patients, 7 were excluded due to not having DCM, 1 was excluded due to having stable DCM since previous decompressive surgery and a further 58 were excluded from statistical analysis of surgical outcome: 12 did not have surgery, 20 did not have baseline mJOA scores documented, 15 did not have postoperative mJOA scores documented, 11 were participating in the RECEDE-Myelopathy trial^34^ or IMAGE DCM trial. Of the remaining 193 patients, 3-month post-operative follow up data were available for 107 patients, 6-month follow up data were available for 96 patients and 12-month follow up data were available for 114 patients (Figure 1).

### Patient Demographics

In total, 57% of patients (142/251) were male, the mean age was 63 (+/- 12) years old, the mean length of symptoms at the first pre-operative clinic appointment was 22 (+/- 24) months and the mean BMI was 29.5 (+/- 6.0). Of the 193 patients whose data was amenable to statistical analysis, 17 patients were graded as having mild, 73 moderate and 103 severe DCM (Table 1). 91.6%, 89.6% and 93.0% of patients had moderate or severe DCM at 3, 6 and 12 month follow up (Table 2).

**Table 1.** Demographics and Surgical Variables*.

Number of Patients	251
Mean age at first clinic appointment	63 +/−12 years
Male	56.6% (142/251)
Body mass index (BMI)	29.5 +/− 6 kg/m^2^
Ethnicity	
White British	76.9% (193/251)
Not reported	14.7% (37/251)
Other background	3.6% (9/251)
Asian Pakistani	1.6% (4/251)
Asian Indian	1.2% (3/251)
Mixed	0.8% (2/251)
Black Caribbean	0.8% (2/251)
Black African	0.4% (1/251)
Tobacco smoking status	
Current smoker	31.8% (76/239)
Former smoker	21.8% (52/239)
Never smoked	36.4% (87/239)
Not reported	10.0% (24/239)
Mild DCM	8.8% (17/193)
Moderate DCM	37.8% (73/193)
Severe DCM	53.4% (103/193)
Median number of spinal levels surgically treated	1
Number of patients who underwent anterior surgery	152
Single vertebral level	99
C3/4	28
C4/5	23
C5/6	38
C6/7	9
C7/T1	1
Two vertebral levels	50
C3/4 & C4/5	14
C3/4 & C5/6	5
C4/5 & C5/6	17
C4/5 & C6/7	2
C5/6 & C6/7	12
More than two vertebral levels	3
Number of patients who underwent posterior surgery	87
Single level	2
C3	1
C4	1
Two vertebral levels	23
C2/3	3
C3/4	7
C4/5	8
C5/6	3
C6/7	2
Three vertebral levels	20
C2/3/4	3
C3/4/5	6
C4/5/6	8
C5/6/7	3
More than three vertebral levels	42
C2-6	1
C2-7	1
C2-T1	1
C3-6	27
C3-7	7
C4-7	2
C3-T1	3
Number of patients who underwent anterior and posterior surgery	0
Median ASA score (n = 234)	3
Patients who received instrumentation^a^	24% (21/87)

Unless otherwise specified, we report mean values, with standard deviation in parentheses. BMI was not documented for 22 patients; tobacco smoking status was not documented for 29 patients; ASA score was not documented for 5 patients.

Percentages in brackets

^a^Percentage given as a proportion of all patients treated by posterior surgery.

**Table 2.** Percentage Breakdown of DCM Severity for Each Follow up Interval.

DCM Severity	3 months	6 months	12 months
Mild	8.4 (9/107)	10.4 (10/96)	7.0 (8/114)
Moderate	36.4 (39/107)	37.5 (36/96)	34.2 (39/114)
Severe	55.2 (59/107)	52.1 (50/96)	58.8 (67/114)

### Patients Managed Surgically

A median number of one spinal level was surgically treated. Median ASA score was three. Patients were grouped into anterior and posterior surgery prior to analysis. 152 patients were treated with an anterior approach (63.6%) while 87 patients were treated with a posterior approach (36.4%).

Of patients treated with an anterior approach, 99 patients were treated for a single level, 50 were treated for 2 levels and 3 were treated for more than 2 spinal levels. For anterior surgery, the most commonly treated single level was C5/6 (38%, 38/99), followed by C3/4 (28%, 28/99), C4/5 (23%, 23/99), and C6/7 (9.1%, 9/99). Other levels were less commonly operated: C7/T1 (1%, 1/99).

Of the 87 patients treated with a posterior approach, only 2 underwent surgery at a single spinal level, while the remaining 85 were treated at multiple levels. Specifically, 23 patients had surgery at two spinal levels, 20 at three levels, and 42 at more than three levels. The most commonly treated two levels were C4/5 (34.8%, 8/23). For three-level surgeries, C4/5/6 was the most frequent (40%, 8/20). Among those treated at more than three levels, C3-6 was the most commonly operated region (64.3%, 27/42), with other sites being less common.

### Surgical Outcomes

**Figure 2.**
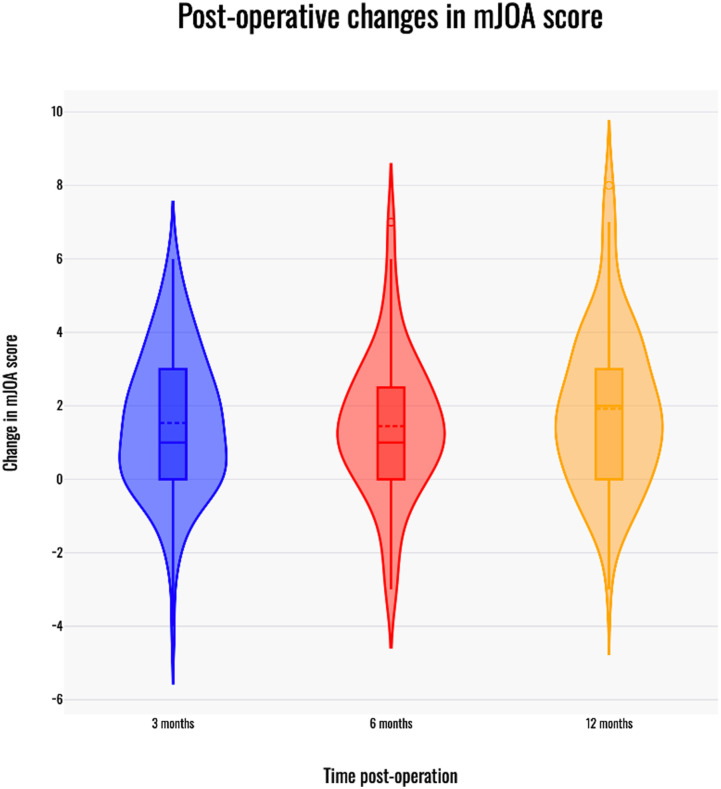
Change in mJOA score with time post-operatively. Violin plots depiciting change in mJOA score at 3, 6 and 12-month follow up. The boxplot indicates median (central solid line), mean (central dotted line) and the interquartile range (outer solid lines) for post-operative change in the mJOA score at each time interval.

There was a significant improvement in the mean total mJOA score by 1.53 points at 3 months (*P* < .001), 1.44 points at 6 months (*P* < 0.001) and 1.92 points at 12 months (*P* < .001) post-operatively compared to pre-operative scores (Table 3). In addition, improvement in the upper extremity motor dysfunction score was significant at 3 months (*P* < .001), 6 months (*P* < .001) and 12 months (*P* < .001). Improvement in the mJOA score was greater than or equal to the minimally important clinical difference for 41% (44/107) of patients at 3-months, 34% (33/96) at 6-months and 43% (49/114) at 12-months (Figure 2).

**Table 3.** Surgical Outcomes^a^.

	Pre-op^b^	Three months Post-op (n = 107)	Pre-op^b^	Six months post-op (n = 96)	Pre-op^b^	Twelve months Post-op (n = 114)
Upper extremity motor dysfunction score	3.28 (1)	3.66 (1.03)^c^	3.32 (1.04)	3.72 (0.95)^c^	3.24 (0.95)	3.87 (0.98)^c^
Lower extremity motor dysfunction score	4.24 (1.11)	4.67 (1.25)	4.39 (1.15)	4.62 (1.17)	4.21 (1.17)	4.65 (1.28)^c^
Sensation score	1.69 (0.63)	2.07 (0.7)	1.65 (0.63)	2.03 (0.60)	1.60 (0.63)	2.08 (0.60)
Sphincter dysfunction score	1.92 (0.72)	2.27 (0.71)	1.97 (0.73)	2.32 (0.61)	1.93 (0.72)	2.30 (0.72)
Total mJOA score	11.12 (2.50)	12.65 (2.81)^c^	11.32 (2.56)	12.77 (2.49)^c^	10.99 (2.42)	12.91 (2.52)^c^
% achieved MCID^d^ (no. of patients)		41 (44)		34 (33)		43 (49)
% whose mJOA score decreased (no. of patients)		9 (10)		9 (11)		11 (13)
% whose mJOA score was unchanged (no. of patients)		24 (26)		16 (15)		15 (17)

^a^Unless otherwise specified, we report mean values (standard deviation) where appropriate.

^b^Individual pre-operative mJOA comparisons are made to 3 months, 6 months and 12 months post-op intervals, respectively, because each comparison considers a separate subset of patients. For example, not all patients who had a 6-months post-op appointment had a 3-months post-op appointment.

^c^*P* < .001.

^d^MCID = minimum clinically important difference.

### Surgical Complications

Intraoperative complications (Table 4) occurred in 11.7% (28/239) of patients. The most common complications were C5 palsies (2.9%, 7/239) and superficial wound infections (2.9%, 7/239).

**Table 4.** Operative Complications.

Complication	% Anterior Surgery	% Posterior Surgery	Total (% of Patients)
C5 palsy	2.6 (4/152)	3.4 (3/87)	2.9 (7/239)
Reperfusion injury	0.7 (1/152)	1.1 (1/87)	0.8 (2/239)
Deep wound infection	0.7 (1/152)	1.1 (1/87)	0.8 (2/239)
Superficial wound infection	2.0 (3/152)	4.6 (4/87)	2.9 (7/239)
Epidural haematoma	0 (0/152)	2.3 (2/87)	0.8 (2/239)
Retropharyngeal haematoma	0.7 (1/152)	0 (0/87)	0.4 (1/239)
DVT	0 (0/152)	1.1 (1/87)	0.4 (1/239)
Dural tear	0.7 (1/152)	0 (0/87)	0.4 (1/239)
Implant failure	0.7 (1/152)	0 (0/87)	0.4 (1/239)
Dysphagia	1.3 (2/152)	0 (0/87)	0.8 (2/239)
Worsening radiculopathy	1.3 (2/152)	0 (0/87)	0.8 (2/239)
Total	10.5 (16/152)	13.8 (12/87)	11.7 (28/239)

### BMI as a Predictor of Surgical Outcomes

There was no significant association between BMI and changes in mJOA scores (Supplemental Figure 1) at 3 months (R^2^ = .0000055, F(1,95) = .00052, *P* = .982) or at 12 months post-surgery (R^2^ = .00045, F(1,93) = 0.042, *P* = .838).

## Discussion

### Summary of Findings

Patients were provided care in accordance with the AO Spine guidelines and subsequent results show significant and clinically meaningful improvements in mJOA score at 3-months, 6-months and 12-months follow-up. These results, along with the complication rate and cohort demographics are comparable to data from international cohorts.

### Findings in Context

The demographics of our cohort are consistent with the literature, with a mean age of 63 and a male preponderance (57%).^24,25^ Our surgical outcomes are comparable to those of a multinational cohort study of 479 patients which showed a significant post-operative improvement in mean mJOA score by 2.36 points at 12 months.^25^ The rate of intraoperative complications in our study was 11.7% (28/239), which is at the lower end of the complication rates reported in the literature, which range from 11 to 38%.^22,24,25,35,36^

The mean preoperative and postoperative mJOA scores were 11.12 & 12.65 for the 3-months follow up, 11.32 & 12.77 for the 6-months follow up and 10.99 & 12.91 for the 12-months follow-up. Of the patients treated surgically, an anterior approach was used for 64% (152/239) patients and a posterior approach was used for 36% (87/239) of patients. In comparison, a North American cohort study^24^ reported an anterior approach in 61% of patients (169/278), a posterior approach in 34% (95/278) and a combined approach in 5% (14/278), whilst a multinational cohort study^25^ reported an anterior approach in 58% (276/478), a posterior approach in 40% (191/478) and a combined approach in 2% (11/478).

The mJOA score was used for assessing patients and their change in functional status over time.^30,37^ Having a dependable predictor for mJOA improvement would prove advantageous in tailoring treatment plans for patients. For example, our analysis of the relationship between BMI and mJOA scores indicated no association, which is in concordance with Wilson et al.^38^ Other unexplored factors may play a more substantial role in predicting early postoperative neurological recovery in DCM patients.

To aid in the interpretation of our results, it should be recognised that little is known about the inter-observer and intra-observer reliability of this particular mJOA score in the English language and the minimum detectable change in this score has been reported as 2.08 points.^32^ A 2020 review describes the overall inter-observer and intra-observer reliability for the mJOA scores as “modest”.^5^ Furthermore, a discrepancy has been reported between improvements in mJOA score and patients’ self-reported health status^39^; 24% of 263 patients whose improvement in mJOA scores achieved the MCID reported either no change or a decline in their overall health.^40^

### Limitations

Our findings are based on the evaluation of a single-surgeon and single-centre experience, with outcome measures limited to the mJOA score and complication rate. Whilst this may limit the generalisability of individual findings (e.g. complication rate), our principal aim was to evaluate implementation of the guidelines in a real-world setting.

Follow-up data was missing in some instances, and only available for up to 12 months postoperatively at the time of the evaluation. Whilst many DCM studies have continued beyond 12 months, little additional recovery is expected after this time point, with most recovery achieved by 6 months. This is reflected in our series, with relatively little change between 6 and 12 months.^41^

## Conclusion

This is the first study to report surgical outcomes for DCM patients following implementation of the AO Spine guidelines. These guidelines are simple to apply, and most patients experienced clinically meaningful improvements in the severity of their myelopathy. Despite its limitations, this study provides reasonable evidence in support of the new guidelines.

## Supplemental Material

**Supplemental Material -** Outcomes for Degenerative Cervical Myelopathy Following Implementation of the AO Spine International Guidelines: A Single-Centre Service Evaluation Supplemental Material for Outcomes for Degenerative Cervical Myelopathy Following Implementation of the AO Spine International Guidelines: A Single-Centre Service Evaluation by Jamie Brannigan, Sundar K. Vellaiyappan, Oliver D. Mowforth, Joseph Magee, Jibin J. Francis, Benjamin M. Davies, and Mark R. Kotter in Global Spine Journal.
