# Supplementary material for: Outcomes for Degenerative Cervical Myelopathy Following Implementation of the AO Spine International Guidelines: A Single-Centre Service Evaluation
Source: Global Spine J. 2024 Nov 10;15(4):2400–8. doi: 10.1177/21925682241301049 (PMC11561916; doi:10.1177/21925682241301049)
Supplement: Supplemental Material - Outcomes for Degenerative Cervical Myelopathy Following Implementation of the AO Spine International Guidelines: A Single-Centre Service Evaluation [file sj-pdf-1-gsj-10.1177_21925682241301049.pdf]

Supplementary figures

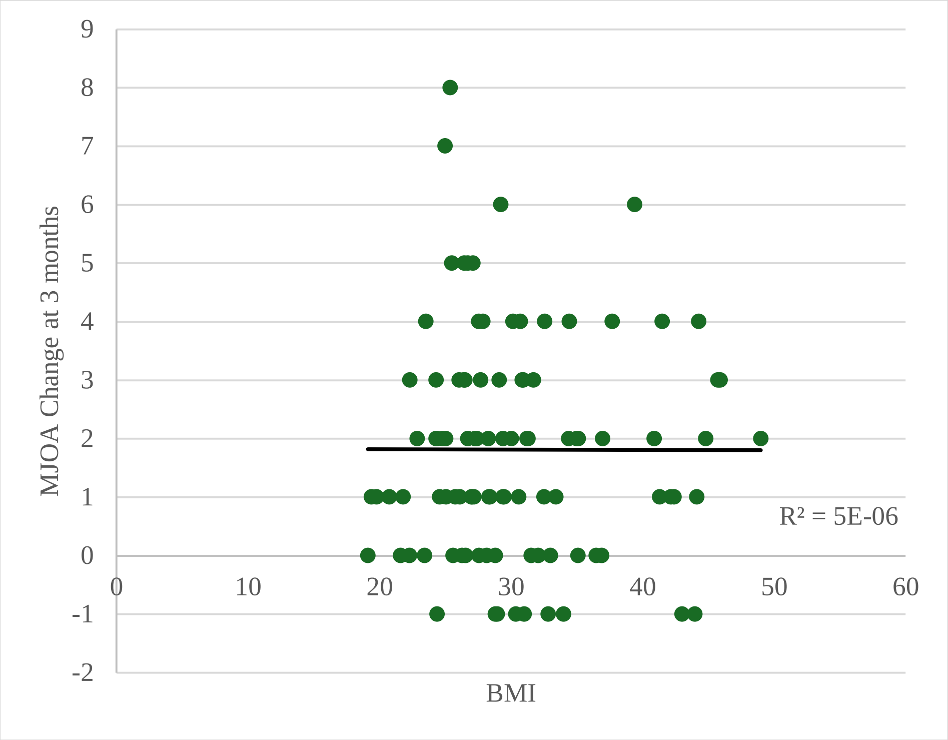

Figure 1a: mJOA change against patients' BMI at 3 months. N=97.

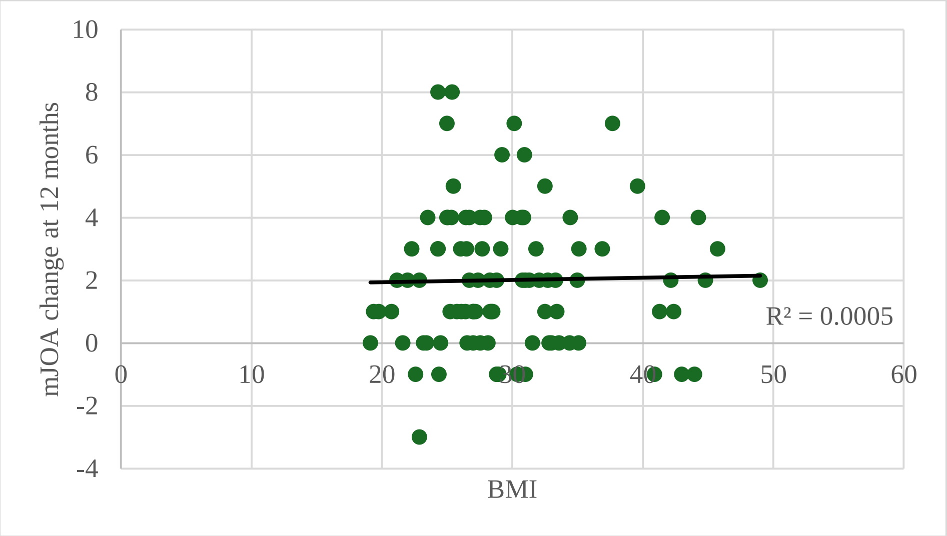

Figure 1b) mJOA change against patients' BMI at 12 months. N=95
